# Ten Simple Rules for a Successful Cross-Disciplinary Collaboration

**DOI:** 10.1371/journal.pcbi.1004214

**Published:** 2015-04-30

**Authors:** Bernhard Knapp, Rémi Bardenet, Miguel O. Bernabeu, Rafel Bordas, Maria Bruna, Ben Calderhead, Jonathan Cooper, Alexander G. Fletcher, Derek Groen, Bram Kuijper, Joanna Lewis, Greg McInerny, Timo Minssen, James Osborne, Verena Paulitschke, Joe Pitt-Francis, Jelena Todoric, Christian A. Yates, David Gavaghan, Charlotte M. Deane

**Affiliations:** 1 Department of Statistics, University of Oxford, Oxford, United Kingdom; 2 Centre for Computational Science, Department of Chemistry, University College London, London, United Kingdom; 3 Centre for Mathematics, Physics and Engineering in the Life Sciences and Experimental Biology, University College London, London, United Kingdom; 4 Department of Computer Science, University of Oxford, Oxford, United Kingdom; 5 Wolfson Centre for Mathematical Biology, Mathematical Institute, University of Oxford, Oxford, United Kingdom; 6 Department of Mathematics, Imperial College London, London, United Kingdom; 7 Department of Genetics, Evolution and Environment, University College London, London, United Kingdom; 8 Centre for Information & Innovation Law, University of Copenhagen, Copenhagen, Denmark; 9 Guest Scholar, Harvard Law School, Cambridge, Massachusetts, United States of America; 10 Visiting Research Fellow, University of Oxford, Oxford, United Kingdom; 11 Department of Dermatology, Medical University of Vienna, Vienna, Austria; 12 Laboratory of Gene Regulation and Signal Transduction, Departments of Pharmacology and Pathology, University of California, California, San Diego, United States of America; 13 Department of Mathematical Sciences, University of Bath, Bath, United Kingdom

## Introduction

Cross-disciplinary collaborations have become an increasingly important part of science. They are seen as key if we are to find solutions to pressing, global-scale societal challenges, including green technologies, sustainable food production, and drug development. Regulators and policy-makers have realized the power of such collaborations, for example, in the 80 billion Euro "Horizon 2020" EU Framework Programme for Research and Innovation. This programme puts special emphasis on “breaking down barriers to create a genuine single market for knowledge, research and innovation” (http://ec.europa.eu/programmes/horizon2020/en/what-horizon-2020).

Cross-disciplinary collaborations are key to all partners in computational biology. On the one hand, for scientists working in theoretical fields such as computer science, mathematics, or statistics, validation of predictions against experimental data is of the utmost importance. On the other hand, experimentalists, such as molecular biologists, geneticists, or clinicians, often want to reduce the number of experiments needed to achieve a certain scientific aim, to obtain insight into processes that are inaccessible using current experimental techniques, or to handle large volumes of data, which are far beyond any human analysis skills.

The synergistic and skilfulcombining ofdifferent disciplines can achieve insight beyond current borders and thereby generate novel solutions to complex problems. The combination of methods and data from different fields can achieve more than the sum of the individual parts could do alone. This applies not only to computational biology but also tomany other academic disciplines.

Initiating and successfully maintaining cross-disciplinary collaborations can be challenging but highly rewarding. In a previous publication in this series, ten simple rules for a successful collaboration were proposed [1]. In the present guide, we go one step further and focus on the specific challenges associated with cross-disciplinary research, from the perspective of the theoretician in particular. As research fellows of the 2020 Science project (http://www.2020science.net) and collaboration partners, we bring broad experience of developing interdisciplinary collaborations. We intend this guide to be for early career computational researchers as well as more senior scientists who are entering a cross-disciplinary setting for the first time. We describe the key benefits, as well as some possible pitfalls, arising from collaborations between scientists with very different backgrounds.

### Rule 1: Enjoy Entering a Completely New Field of Research

Collaborating with scientists from other disciplines is an opportunity to learn about cutting-edge science directly from experts. Make the most of being the novice. No one expects you to know everything about the new field. In particular, there is no pressure to understand everything immediately, so ask the “stupid” questions. Demonstrating your interest and enthusiasm is of much higher value than pretending to know everything already. An interested audience makes information sharing much easier for all partners in a collaboration.

You should prepare for a deluge of new ideas and approaches. It is a good practice to read relevant textbooks and review papers, which your collaborators should be able to recommend, in order to quickly grasp the vocabulary (see Rule 3) and key ideas of the new field. This will make it easier for you to establish a common parlance between you and your collaborators, and allow you to build from there.

You should try to discuss your work with a range of scientists from complementary fields. As well as getting feedback, this can help you identify new collaborative opportunities. Remember that contacts that do not lead directly to collaborations can still prove useful later in your career.

### Rule 2: Go to the Wet Lab

It is vitally important to understand where specific data sets come from. Just like mathematical and computational models, experiments have their own in-built assumptions, strengths, and weaknesses that you need to understand. What was the exact process of data collection? How many experiments can be performed in a given timeframe and how much do they cost? What were the constraints that led to the design of the experiments—how will you include this in your interpretation? If you plan to use the resulting data for model calibration or parameter fitting then try to obtain sufficient information to reproduce the experiment in silico. Papers in different domains have different perspectives and might not contain the data you are looking for in sufficient detail. Visiting the lab in person is often the most efficient way to get the information you need. A good understanding of the experimental setup might also suggest appropriate testcases for the computational studies. Try to talk to both the junior and senior scientists in the lab as they may give you different perspectives.

There are social, as well as scientific, reasons for understanding life in the wet lab. As a computational scientist, it is easy to underestimate the commitment and resources necessary to acquire experimental data (see rule 4). Visiting a lab, and taking an interest in data collection, is a way of acknowledging your colleagues’ effort and the value of their data and expertise.

### Rule 3: Different Fields Have Different Terminologies: Learn the Language

Science is full of subcultures using diverse and evolving jargon. Forming a successful cross-disciplinary relationship requires that you fully understand your collaboration partner. From classification schemes and methods to journals and research philosophy, it can be hard enough keeping up with developments in your own field, let alone others. For instance, neologisms can be ubiquitous in computational and biological sciences, where new terminology continually emerges from new methods, tools, and knowledge. Learn the other field’s jargon early on in the collaboration and ask basic questions about the meanings of words.

For example:

- Ambiguity: “Model” is probably the most ambiguous word in science. Mathematical, statistical, experimental, observational, theoretical, computational, analytical, verbal, legal, mental, graphical, geometrical, structural, and workflow models all have different meanings. Almost every field will have its own interpretation of “model” and the semantics differ significantly.
- Synonyms: For example, removing entities above and below certain thresholds is termed “positive and negative selection” in immunology, while it is called “band-pass filter” in signal transduction.

Context often matters, so try to understand nuances in the use of terms. It can be beneficial to build up a technical glossary. Evaluate your understanding by presenting it back to new colleagues and observe where your rudimentary understanding needs more work.

Finally, agree on a joint nomenclature with your collaborators early in the project. Write equations and code in a consistent manner, standardise data formats, and use consistent style schemes in figures. Then talk through your outputs to discuss your collaborators’ understanding and involvement. A good relationship is based on mutually understandable communication.

### Rule 4: Different Fields Move at Different Speeds: Do not Become Impatient

A huge variety of cultures and expectations regarding research and subsequent publication exist in different scientific disciplines. However, these differences can lead to stress when embarking upon multidisciplinary collaborations, unless they are acknowledged and effectively communicated at an early stage. It is important to accept the different pace of different fields, communicate well, and be patient.

Research in experimental biology, for example, often involves long and arduous experiments, taking perhaps months or even years to complete. Animals or tissues may need to be grown, and weekends or nights spent in the lab tending to cell cultures and repeating experiments may be necessary. Some projects generate publications and co-authorships several years after a theoretician may have actually performed their in silico contribution to the work. Vice versa, computational aspects often involve more than simply pressing a button and computational resources may be limited.

Do not make assumptions about how hard fellow collaborators are working based on how long they take to get back to you with results. Here, communication is of particular importance.

Similarly, journals in different disciplines might have different periods of time from submission to publication. This can have knock-on effects when demonstrating your research output (see rule 5).

Early communication of how long your part of the work is likely to take and why this amount of time is needed will help your collaboration to run more smoothly.

### Rule 5: Different Fields Have Different Reward Models: Know What You Can Expect

It is important to recognise that the publication culture in the life sciences, and in experimental biology particularly, differs from that of the theoretical sciences. Such differences can include:

- Publication speed varies greatly. In experimental biology, publishing often takes several years, while certain theoretical papers can be published in a much shorter timescale (see also rule 4).
- Metrics, such as the impact factor (IF), are used by many organisations to evaluate your research [2,3]. Be aware that different fields have different impact factor scales. The journal impact factors mainly depend on the average length of reference lists in the field. For example, a journal with an impact factor of 3 in mathematics (295 journals, median IF 0.57, maximum IF 3.57) might be more prestigious than a journal with an impact factor of 30 in cell biology (184 journals; median IF 3.2; maximum IF 37.16) (based on Journal Citation Reports of Thomson Reuters, version 2012).
- In some fields, such as information technology, it can be the norm to publish new research in peer-reviewed conference proceedings instead of journals.
- The preferred ordering of authors on a manuscript may also depend strongly on the academic environment. The first author might be the scientist who contributed most or whose surname comes first alphabetically. The last author may be the principal investigator, the author who contributed least, or the author with the last surname alphabetically. The corresponding author can be seen as “in charge of the paper,” the principal investigator, or the person who volunteered for dealing with the correspondence.
- In some areas of biology, large consortia of authors are needed to conduct research. In some theoretical fields, people tend to publish with fewer authors. Thus, the definition of a “significant” contribution to a manuscript might differ markedly.

It is important to be aware of these differences. Make sure that you discuss all these issues early with your collaborators to avoid misunderstandings and frustration. A well-planned publication strategy is fundamental in order to fulfil everyone’s expectations and accommodate the potential mismatch of timescales of theoretical/experimental work (see rule 4).

Does your field and that of your partner value less frequent, higher impact publications or a series of smaller publications? One option is to start with methodological papers (both theoretical and experimental) while final publications describing the major breakthrough and how all the components are brought together could follow. Early methodological papers should already highlight the benefits of collaborating, e.g., theoretical work with experimentally sound assumptions and parameters, experimental work with solid data analytics. It is advisable to design initial publications without forgetting the greater scope of the collaboration. However, be aware that preceding papers might weaken your main publication if they anticipate parts of the results.

### Rule 6: What Different Fields Mean by “Data”

Be prepared that scientists with experimental backgrounds might not have the same structured view on data and terminologies (see also rule 3) as you have. For scientists with a background in computer science, the lowest level of data organisation might be a spreadsheet where each column and row is well defined. For scientists with non-technical backgrounds, such a spreadsheet might represent the highest form of data organisation.

Whenever possible, ask your collaboration partner for a standardized data format. Good guides on how to share data can be found in [4,5]. Always favour electronic forms of data and always keep a copy of the original file. You might also consider writing minutes about meetings and data specifications to avoid later misunderstandings.

Do not blindly trust experimental data. Always perform “sanity checks” on the data you receive (graphs, frequency tables, mutually exclusive data, unnatural distributions, etc.). This will help you to see if you have interpreted the data correctly and allow you to ask questions if you have any doubts.

### Rule 7: Assess the Advantages and Disadvantages of Service Work

Theoretical scientists can be a huge asset to multidisciplinary projects by providing experimentalists with computational tools to gather data, predictive methods, and advanced statistical modelling. Theoretical scientists often do substantial amounts of “service work” in a collaborative project, for example, by maintaining computer infrastructure and databases, keeping their in-house code base up-to-date, and statistically analysing data.

Service work is often an excellent way to establish a collaboration, get the partners to trust in your ability and expertise, and learn enough about other disciplines to start making direct contributions, whilst, at the same time, co-authoring high-quality publications. Service work will show that you take the collaboration seriously and help you to establish a reputation as a reliable and analytically keen scientist who delivers fast, structured, and correct results.

Nonetheless, service work is also risky, as it may take more time than you anticipated. Therefore, make sure to evaluate the amount of service work on a regular basis and be clear with your collaborators about what you expect in return before engaging in service work. To gain more insight into the “cost” of service work, keep a record of the amount of time spent on service tasks. This not only prevents your collaborators from treating your contributions lightly but also gives you a clear idea as to whether it is worthwhile to engage in such tasks and/or take on new ones.

Crucial to minimizing the service “'load” is to make it easy to delegate tasks to others. When starting to develop analytical tools for others (whether software or mathematics), always take the perspective that these tools won't primarily be used by you, but by other collaborators. Hence, making your tools user-friendly, for example, by providing illustrative examples and documenting your code extensively [6], is essential.

### Rule 8: Create and Manage Structural Bonds

Cross-disciplinary collaborations require structural bonds between the collaborators. It is only possible to break the silos of scientific disciplines and to become truly cross-disciplinary if a proper framework for scientific exchange is established. This can include regular meetings, workshops, symposia, attendance of each other’s group meetings, and co-teaching of courses. However, to keep your collaboration efficient, be careful about imposing too many obligations. While it is often necessary to leave the “comfort zone”of scientific disciplines, it is equally important not to frustrate your collaboration partners with too many details not relevant to their endeavours. Therefore, keep the number of meetings at a reasonable level and set clear agendas.

Moreover, establishing these bonds often requires financial support, which can be achieved in different ways. For the initial setup phase, seed funding schemes maybe a useful resource. On the basis of these initial bonds, applications for larger grants can be submitted collaboratively. Many funding bodies offer special calls for cross-disciplinary research or favour cross-disciplinary proposals for both national and international settings. Examples include the "Horizon 2020" EU Framework (http://ec.europa.eu/programmes/horizon2020/en/what-horizon-2020), the Human Frontiers in Science Program (http://www.hfsp.org/), the US NSF/BIO–UK BBSRC Lead Agency Pilot (http://www.bbsrc.ac.uk/funding/internationalfunding/nsfbio-lead-agency-pilot.aspx), or internal projects created to achieve inter-faculty cooperation within a university.

Once funding is secured, shared PhD students and postdocs can further strengthen bonds between collaboration partners. Shared supervision rewards with constant knowledge exchange, shared publications, and an interdisciplinary training for all. However, it is also important to protect junior scientists from “getting lost in cross-disciplinary collaboration.”In particular, there is a risk that they fall between two stools in their attempts to comply with the expectations and advice of two or more supervisors from completely different fields.

### Rule 9: Recognise When Things Are Not Working Well

Unlike a marriage, collaborations are not necessarily intended to be continuous and permanent. A pragmatic approach can favour both parties. If major problems arise that cannot be solved after a couple of attempts, there are several possible next steps:

1. Ostrich approach: Pretend that nothing has happened and hope that normality will soon be restored. We strongly advise against this tactic as it might lead to more frustration and potential damage to the relationship.
2. Pause: Sometimes, one of the collaborators may find it harder than expected to deliver the agreed results or is overwhelmed with other work-related duties. If your collaborator has trouble with their side of the workload, it is often better to introduce a pause in the collaboration than to exert pressure on them. Deliberate pauses take pressure off your collaborations, and can save potential frustration on your side as well.
3. Search for alternatives: Collaborations do not need to be exclusive. If you prioritise the collective work more highly than your collaborator, you might consider establishing a fresh collaboration with someone else who also values the work more. But be aware of your actions and the consequences they might have on your current collaboration.
4. End a collaboration: If a collaboration has become unworkable or reached a natural terminus, it may be best to make a clean cut and end the collaboration once and for all.

Many failures in collaborations could have been avoided by an early, proactive approach on arising problems. In cross-disciplinary settings, it could just be a problem of understanding (rule 3), impatience (rule 4), or lack of reward (rule 5). If you decide to end a collaboration, make sure to keep a working relationship with your former collaborator. This will allow you to properly handle existing structural bonds (rule 8) and allow you to potentially initiate other collaborations with the same partner.

### Rule 10: Be Synergistic

Probably the most important quality of collaborations is the mutual gain that emerges. This usually works best when scientists with different, but complementary, skills decide to work together. One example might be the application of a novel, high-throughput computer algorithm to a vast quantity of experimental data. While the algorithm by itself might be brilliant, and publishable by benchmarking it on a publicly available dataset, it will shine even more when applied to a huge, unpublished dataset. At the same time, the huge dataset could be analysed by standard, semi-manual methods. This might also be publishable, but would take a long time and essential insights may be missed, which the novel algorithm would have delivered. Only by combining contributions from both sides does the work become more than the sum of its parts, achieving useful things for each partner and enabling insights that would not have been possible for either alone.

Initiating and nurturing successfully synergistic relationships is an important and valuable skill. The success of an interdisciplinary collaboration depends on any number of factors, including which partner approached which and over what timescale each party envisages collaboration. One very important property for a long-term, effective, and mutually beneficial relationship is that both sides should feel they are winners during and, especially, after a project (see also rule 5). An outstanding book on how such win/win situations can be achieved is Fisher, Ury, and Patton’s *Getting to Yes*: *Negotiating Agreement Without Giving In* [7]: their advice includes inventing options for mutual gain, making sure to always give enough credit to partners, and caring for their interests as you would for your own. Then you will establish a truly successful and synergistic collaboration.
